# Surveillance of Gallbladder Polyps: A Literature Review

**DOI:** 10.7759/cureus.16113

**Published:** 2021-07-02

**Authors:** Deepak P Kalbi, Anusha Bapatla, Ahmed J Chaudhary, Sifullah Bashar, Sana Iqbal

**Affiliations:** 1 Internal Medicine, Detroit Medical Center/Sinai Grace Hospital/Wayne State University, Detroit, USA; 2 Internal Medicine, California Institute of Behavioral Neurosciences & Psychology, Fairfield, USA; 3 Internal Medicine, Detroit Medical Center, Detroit, USA

**Keywords:** gallbladder polyps, benign, malignant polyps, surveillance, gallbladder pseudopolyps

## Abstract

Little has been documented in existing literature regarding incidentally found gallbladder (GB) polyps. These clinically asymptomatic lesions are mostly benign, with only 5% progressing to malignancy. GB cancer, although rare, presents as an end-stage incurable disease. According to the current guidelines, cholecystectomy is recommended for polyps >10 mm in size for a better outcome. Thus, it is essential to know the clinical picture, surveillance, and treatment of these polyps earlier in the course of the disease to avoid the advancement of polyps to malignancy. This paper discusses the signs and symptoms, surveillance, treatment, and prognosis of GB polyps.

## Introduction and background

Protrusion of the elevated, sessile or pedunculate gallbladder (GB) mucosa into the lumen is defined as a GB polyp [1]. Often found incidentally on imaging, or after cholecystectomy in patients with acute cholecystitis or biliary colic [2], these benign lesions are mostly clinically insignificant, with progression to malignancy being the primary dilemma. GB polyps are classified either as pseudopolyps that are benign in nature or true polyps that may have malignant potential. Only 5% are reported as a true polyp. It is important to diagnose those few malignant polyps to undergo definitive treatment and ensure long-term survival. Clinically, polyps are either asymptomatic or may present as a picture of acute cholecystitis when it obstructs the cystic ducts or as cholangitis when its fragments occlude the bile flow [3].

GB adenocarcinoma is a rare entry with a grave prognosis if diagnosed at the end-stage. It is the 20th most common cancer in the world [3]. According to the American Joint Committee on Cancer (AJCC), survival and prognosis are based on the staging of the disease with an 80% five-year survival rate in patients with stage 0 carcinoma in situ lesions and 2% with stage 4b disease [3].

GB polyps don’t always require surgery, as pseudopolyps possess no malignant potential. The surgery itself is associated with several complications such as bile duct injury, bile leak, and damage to intra-abdominal structures [3]. Cholecystectomy is the ultimate treatment for “true” GB polyps, and laparoscopic cholecystectomy is the treatment of choice, whereas, pseudopolyps don’t require any further surveillance, interventional treatment, or follow-up.

This paper differentiates between pseudopolyps and high-risk true GB polyps, their detection, and management. True GB polyps need to undergo surveillance for early detection of worrisome GB adenocarcinoma for assuring long-term survival.

## Review

Incidence

Found mostly on abdominal ultrasound, the prevalence of GB polyps is 0.3%-9.5% [1]. Found mostly in men with a male to female ratio of 1.15:1, they are commonly detected at the age of 49 [2]. Almost 70% of the polyps are pseudopolyps. Approximately 178100 cases of GB carcinomas are reported every year with the highest incidence seen in South America and Asia. It is relatively uncommon in North America and the USA [3].

Types

In the 1970s, benign polyps were classified by Christensen and Ishak as pseudopolyps and epithelial and mesenchymal tumors. Pseudopolyps include cholesterol polyps, inflammatory polyps, cholesterolosis, and hyperplastic polyps. Epithelial tumors include adenomas, whereas mesenchymal tumors include fibroma, lipoma, and hemangioma [2].

Benign GB polyps are mostly asymptomatic, with cholesterol polyps being the most common type, whereas the incidence of malignant adenocarcinoma is only 0.4% [4].

Cholesterol Polyps

Cholesterol polyps are the most common type of GB polyps with an incidence rate of 60%-90%. These are small, multiple polyps with a size less than 10 mm in diameter and have no malignant potential. Metabolic syndrome plays a role in the development of cholesterol polyps. 

Inflammatory Polyps

With an incidence of 10%, these polyps are benign with a size less than 10 mm in diameter. The pathophysiology of these polyps is associated with local epithelial proliferation secondary to chronic inflammation; these are often associated with chronic cholecystitis [2,5].

GB Adenomas

These rare, benign tumors are thought to have a premalignant potential. These generally solitary lesions are 5-20 mm in diameter. They are usually asymptomatic and detected incidentally on imaging or in cholecystectomy specimens. A few may become symptomatic via cystic duct obstruction or association with symptomatic gallstones. 

Macroscopically, it may have a sessile, pedunculated, or polypoid shape. Microscopically, it may have a tubular, papillary, or tubulopapillary configuration with the tubular type being the most common on histopathology reports.

Adenomyomatosis

Thought to be a precancerous lesion, they constitute 25% of GB polyps. Mostly found as a solitary polyp, they are localized in the GB fundus, and their incidence increases with age [2].

Clinical presentation

GB polyps are usually symptomatic and found incidentally on ultrasound. Occasionally, they have nonspecific gastric symptoms such as nausea, vomiting, and right hypochondriac pain due to intermittent obstruction caused by fragments of cholesterol detached from GB mucosa. Rarely, some large polyps may obstruct the cystic duct causing acute cholecystitis or obstructive jaundice. Benign and malignant polyps have a similar clinical presentation. According to a study, 64% of the polyps are found incidentally on workup done for an unrelated illness, 23% presented with abdominal symptoms, and 13% had raised liver function tests.

Risk factors associated with GB polyps

There is no significant study identifying the risk factors for the development of GB polyps. Fat metabolism has been linked with the pathophysiology of gallbladder polyps. However, no significant relationship has been found between the formation of polyps and age, gender, obesity, and diabetes mellitus [2].

Factors for GB Polyp Malignancy

Generally, polyps less than 10 mm are considered benign. However, studies have shown malignant polyps of less than 10 mm. Thus, it is important to identify the risk factors associated with polyp malignancy for early detection and appropriate malignancy (Table 1).

**Table 1:** Summary of risk factors for malignant GB polyps GB: gallbladder

Risk factors	Association of the risk factor with gallbladder carcinoma
Age	Per Bhatt et al, the probability of carcinoma is found to be 20.7% in patients aged over 50 who have polyps less than 10 mm in diameter [5].
Ethnicity	The risk of malignancy is 13 times higher in Indian and Asian ethnicity as compared to Caucasians [3].
Gallstones	Association between the gallstone and gastric carcinoma is not well-proven.
Size	Size more than 10 cm is considered as the cutoff for suspicion of high risk of malignancy [6].
Shape	Solitary polyps are more malignant than multiple polyps. The probability of malignant is 24.8% in solitary sessile polyp and cholecystectomy is recommended [3,6].
Primary sclerosing cholangitis	According to a case series of 4 patients with primary sclerosing cholangitis and gallbladder polyps, all were found malignant regardless of the size of the polyp [3].
Tumors markers	No positive linear association has been found between the tumor markers and the risk of malignancy [3].

Number of Polyps

Few studies suggest solitary polyps are more malignant than multiple polyps but this is controversial [2]. Malignancy should be considered in solitary polyps in combination with other risk factors of malignancy. But further research is necessary as there is no clear evidence regarding malignant potential in solitary versus multiple polyps and management based on the number [3,6].

Shape

Polyps with sessile morphology have a more malignant risk potential than pedunculated polyps. The probability of malignant is 24.8% in solitary sessile polyp and cholecystectomy is recommended [3,6]. According to ESGAR guidelines, cholecystectomy is recommended for all sessile polyps having a diameter of 6-9 mm [3].

Size (Diameter of the Polyp)

Generally, 10 mm size is the cut-off value for suspecting malignancy in polyps. However, the diameter of the polyp shouldn’t be considered a safety factor. Thus cholecystectomy is considered for a 6 mm size polyp, which may be multiple or solitary or sessile or pedunculate [2].

Age

The incidence of GB carcinoma increases in the fifth and sixth decade of life. According to Bhatt et al., the probability of carcinoma is found to be 20.7% in patients aged over 50 who have polyps less than 10 mm in diameter [5].

Ethnicity

The risk of malignancy is 13 times higher in Indian and Asian ethnicity as compared to Caucasians [3]. The evidence of Indian ethnicity is so compelling that according to current guidelines, patients with Indian ethnicity having polyps of 6-9 mm are recommended to undergo cholecystectomy to avoid a grave prognosis of GB carcinoma.

Gallstones

The association between gallstones and the risk of malignancy in GB polyps is of low quality. There is no strong evidence suggesting cholecystectomy for the concurrent presence of gallstones and polyps. However, GB polyp should be evaluated more technically in the presence of stones, as gallstones hinder the evaluation of polyps on ultrasound.

Primary Sclerosing Cholangitis (PSC)

Primary sclerosis cholangitis is a strong risk factor for malignancy in GB polyps. Polyps are usually malignant in patients with primary sclerosis cholangitis. According to a case series of four patients with PSC and gallbladder polyps, all were found malignant regardless of the size of the polyp [3]. Regardless of the size and shape of the polyp, cholecystectomy is recommended.

Tumors Markers

No positive linear association has been found between the tumor markers and the risk of malignancy. Perhaps CA19-9 levels were raised in 4.9% of benign tumors and carcinoembryonic antigen (CEA) levels were raised in 5.7% of the benign tumors and 2.7% of malignant polyps [3].

Imaging techniques in surveillance of GB polyps

Radiological imaging plays a vital role in diagnosing GB polyps and helps one in deciding the mode of intervention and follow-up schedule. Imaging techniques differentiate polyps from other GB pathologies. It also helps us differentiate between pseudopolyps and true polyps, which have different management strategies based on malignant potential. Ultrasonography, computed tomography (CT), and magnetic resonance imaging (MRI) play a pivotal role in the surveillance of polyps. Each modality has its pros and cons, which are discussed below.

Trans-Abdominal Ultrasonography

A trans-abdominal ultrasound is considered one of the best modalities in diagnosing GB polyps due to its low cost and better sensitivity and specificity. It includes a conventional ultrasound (CUS), high-resolution ultrasonography (USG) (HRUS), three-dimensional ultrasound, and contrast-enhanced ultrasound.

GB polyps appear as a fixed hyperechoic mass protruding into the lumen of the gallbladder with or without acoustic shadow. Generally considered a first-line investigation in diagnosing polyps, it cannot detect polyps less than size 5 mm and fails to differentiate between a benign and a malignant polyp. A cholesterol polyp can be distinguished from adenoma or adenocarcinoma by the similar echogenicity to the gallbladder wall and no shadow cone. An impacted gallstone can easily be confused with a polyp on CUS. The differentiation between benign and malignant polyp is made on the size of the polyp, which isn’t sufficient [7-8].

Ultrasound is operator-dependent and may be affected by the body mass index (BMI) of the patient, particularly in those with truncal obesity. CUS is a low-frequency modality that has a sensitivity of 50%-90% and a specificity of 71%-98% in diagnosing gallbladder polyps. The same study identified that CUS has a sensitivity of 47%-67% and specificity of 36%-100% for diagnosing gallbladder malignancy [9].

High-Resolution Ultrasound

HRUS is a better modality in identifying the polyps by using high-frequency waves. A non-invasive technique, HRUS has more accuracy in identifying and staging neoplastic polyps and gallbladder cancer [10].

Jang et al. demonstrated the comparison between the accuracies of high-resolution USG (HRUS), Endoscopic Ultrasound (EUS), and CT scan in diagnosing and staging the gallbladder polyps and cancer in 144 patients with polyps greater than 1 cm in diameter [11]. Results showed that HRUS is the most sensitive of all in diagnosing the neoplastic lesions of the gallbladder. However, the study is biased as patients with polyp more than 10 mm were considered.

Three-Dimensional Ultrasound

It is an evolutionary modality with no operator dependency. According to a study, 3D ultrasonography cannot detect polyps with a size of less than 4 mm. It has an 89% positive predictive value in 80 patients who had polyps viewed by 2D ultrasonography and a specificity of 86% [12].

Contrast-Enhanced Ultrasonography

Contrast-enhanced ultrasonography allows differentiating the benign pathologies of the GB from malignant tumors. According to Hattori et al., tumors were classified as benign or malignant based on the vascularity patterns, which were classified as linear, scattered, diffused, or branched. Diffused and branched vascularity patterns were indicative of carcinoma with 100% sensitivity and 76.9%specificity [13]. In GB cancer staining of the tumors, it is continuous whereas it is scattered in benign lesions.

Endoscopic Ultrasound

EUS uses a high-frequency transducer, thus it provides better imaging. According to a study, EUS has a sensitivity of 67%-86% and specificity of 84%-91% as compared to CUS. EUS is considered the next step in workup after CUS. EUS is recommended for differential diagnosis of polypoid lesions when USG shows no signs of benign polyps. According to Sugiyama et al., EUS was more accurate in differentiating malignancy from benign tumors than CUS (97% vs 76%). A tiny echogenic spot or an aggregation of echogenic spots indicate cholesterol polyps or adenomyomatosis. The absence of these indicates an adenoma or adenocarcinoma of the GB [14].

EUS is an invasive procedure that carries the risk of bleeding and upper GI perforation.

EUS Scoring System

The EUS scoring system has been identified to calculate the risk of malignancy. The formula for scoring is (maximum diameter in mm)+(internal echo pattern score, where heterogeneous = 4, homogeneous = 0) + (hyperechoic spot score; where presence = - 5, absence = 0). If the score is higher than 12, the sensitivity, specificity, and accuracy for malignancy of polyps if 78%, 83%, and 83% respectively [15].

CT Scan

A CT scan is not useful for detecting polyps less than 10 mm in diameter. However, it is used for staging large, suspicious malignant tumors. CT generally shows polypoid GB carcinoma as an enhancing, intraluminal tissue mass denser than surrounding bile [2].

Magnetic Resonance Imaging

Scarce literature is available for determining the role of MRI in evaluating GB polyps. Malignant lesions show early and prolong enhancements, whereas benign lesion shows early enhancement with subsequent washout [2].

Other Modalities

Positive emission tomography showed promising results in differentiating benign tumors from malignant ones. However, more research is required for determining its efficacy.

Percutaneous trans-hepatic fine-needle aspiration (FNA) and percutaneous cholecystoscopy are invasive procedures that are time-consuming and poorly tolerated by patients.

Management

The major influencing factor in the management of polyps is the size of the lesion on radiological imaging. Larger polyps with a size greater than 10 mm are considered malignant. According to the current guidelines of the European Society of Gastrointestinal and Abdominal Radiology (ESGAR), it is recommended that polyps of 10 mm or greater should undergo cholecystectomy. However, there are a majority of malignant polyps that deviate from this cut-off value and are less than 10 mm in diameter [6,9]. Malignant polyps of >1 cm, <1 cm, and <5 cm constitute 8.5%, 1.2%, and 0% of gallbladder polyps [9]. Bhatt et al. also demonstrated that the probability of malignancy under 4.15 mm was 0% [6].

Several studies have suggesting to lower the cuts off value for the indication of cholecystectomy in case polyps to be 6 mm [1]. Offering cholecystectomy to patients with polyps less than 10 mm eliminates the risk of malignancy. However, it will put patients to unnecessary operative complications, as all the polyps are not malignant under 10 mm.

There are no definitive guidelines for the follow-up of polyps based on size. Current guidelines by ESGAR recommend that patients with polyps 6-9 mm should undergo follow-up more frequently than those with polyps less than 6 mm. A single case report showed the development of a tiny 5 mm GB polyp into a 20 cm carcinoma over two years [16]. Thus it is recommended that all the patients with polyps 4-10 mm in diameter should be followed up extensively, as there is a significant number of malignant polyps between 4-6 mm.

**Figure 1:**
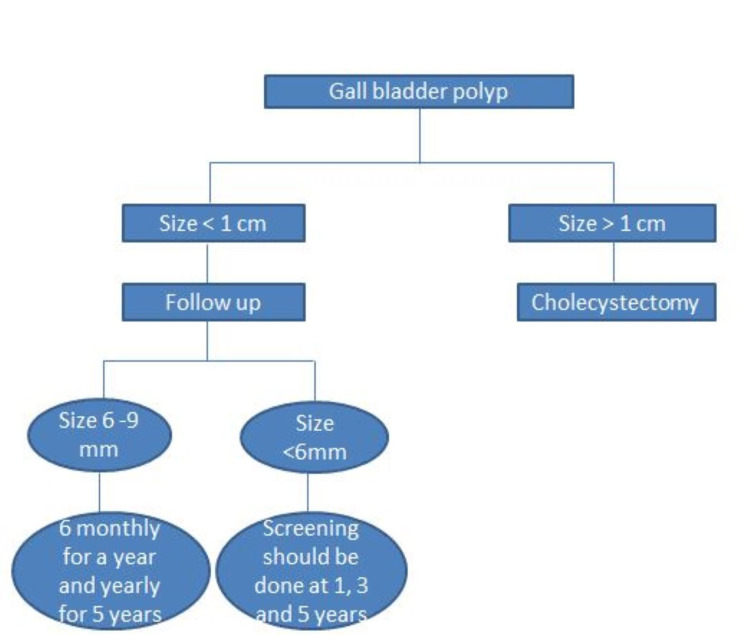
Management approach based on the size (diameter) of the GB polyp GB: gallbladder [9,17]

In a study by Babu et al., it is recommended that polyps of 5-10 mm should undergo surveillance and follow-up six-monthly for a year [9]. ESGAR guidelines suggest that after two six-monthly scans for polyps of 6-9 mm, there should be annual screening for five years. For polyps less than 6 mm, screening should be carried out at one, three, and five years. However, if other risk factors of malignancy are present, the patient must undergo extensive surveillance than those for polyps 6-9 mm with no risk factors [9]. According to Babu et al., only 7.6% of polyps increased in size, and Bhatt et al. also found that only 7% of polyps showed growth. The management approach based on size are illustrated in Figure 1.

Laparoscopic cholecystectomy is done if the serosa is not involved. In the case of serosal involvement, open cholecystectomy is done with resection of nearby liver segments. Cholecystectomy is recommended in patients with age >60 years, fast-growing polyps with size >10 mm, sessile and wide-based polyps with long pedicels, and polyps of gallbladder infundibulum. Young patients who are either asymptomatic or have polyps less than 10 mm only need follow-up with ultrasound six-monthly [2,17].

## Conclusions

GB polyps are the most common surgical vesicular pathology found incidentally on ultrasound or in the specimen of cholecystectomy after an episode of acute or chronic cholecystitis. The majority of the patients are asymptomatic. Ultrasound is considered the best diagnostic modality for identifying polyps. Most polyps are benign; malignant polyps are found in a few cases. Due to the grave prognosis of GB cancer, surveillance is recommended for early detection, prompt treatment, and a better prognosis. Six monthly follow-ups are recommended for patients with polyps 6-9 mm in size and prompt cholecystectomy should be done if the polyps are greater than 10 mm in diameter. Lesions that demonstrate growth, vascularity, invasion, or are symptomatic require cholecystectomy.
